# Photosynthetic pigments in developing seeds of *Acer platanoides* and *Acer pseudoplatanus*

**DOI:** 10.1038/s41598-026-44414-7

**Published:** 2026-03-21

**Authors:** Amir Mohammad Mokhtari, Natalia Wojciechowska, Andrzej Kowalski, Ewa Marzena Kalemba

**Affiliations:** 1 Institute of Dendrology, Polish Academy of Sciences, Ul. Parkowa 5, 62-035 Kórnik, Poland; 2 Department of General Botany, Institute of Experimental Biology, Faculty of Biology, Adam Mickiewicz University, Uniwersytetu Poznańskiego 6, Poznań, 61-614, Poland

**Keywords:** Chlorophyll, Embryogenesis, Orthodox, Recalcitrant, Seed morphogenesis, Seed maturation

## Abstract

Photosynthetic pigments were investigated in developing and dried seeds of desiccation-tolerant (orthodox) *Acer platanoides* and desiccation-sensitive (recalcitrant) *Acer pseudoplatanus*. The seeds of both *Acer* species contained higher levels of chlorophyll *a* (Chl *a*), particularly in cotyledons, than chlorophyll *b* (Chl *b*). Chl *a* and Chl *b* peaked during seed morphogenesis, then declined. The decrease was greater in *A. platanoides* (eight-fold) than in *A. pseudoplatanus* (three-fold) seeds. The total chlorophyll content was greater in the *A. pseudoplatanus* seeds only for three weeks, before morphogenesis began in the *A. platanoides* seeds. Photosystem II activity suggested that photosynthesis might be more active in *A. pseudoplatanus* at the initial and final stages of seed development. The carotenoid (Car) content increased during the initial stages of *A. pseudoplatanus* embryo development. The Car/Chl ratio indicated a significant contribution of Car to the pigment pool during the initial stages of development and drying in *Acer* seeds. The chlorophyll autofluorescence signal was irregular and diffuse in the embryonic axes of mature *Acer* seeds; however, a spherical and compact autofluorescence signal was reported in *A. platanoides* cotyledons. Differences in photosynthetic pigments are discussed in relation to seed orthodoxy and recalcitrance, as potentially influencing the longevity variations associated with seed categories.

**Supplementary Information:**

The online version contains supplementary material available at 10.1038/s41598-026-44414-7.

## Introduction

Chlorophyll is a green pigment present in two distinct forms in higher plants: chlorophyll *a* (Chl *a*) and *b* (Chl *b*). These pigments are the major photoacceptors in green plants, enabling the plants to absorb energy from light and initiate photosynthesis. The ratio of Chl *a* to Chl *b* is typically 3:1 in plants, except under shaded conditions, when relatively high levels of chlorophyll b are detected^1^. Chlorophylls are present within the chloroplast and are specifically bound to the inner membrane of this organelle. In the plant kingdom, photosynthesis occurs in leaves, stems, flowers, fruits, and seeds and, exceptionally, in roots^2^.

Seeds represent the most important form of plant reproduction and play an important role in their spread. Those exhibiting a green color are termed chloroembryos, whereas chlorophyllous cells refer to cells with intact chloroplasts^3^. Chloroembryos are considered unique photosynthesis systems. The reason why some chloroembryos are capable of performing photosynthesis remains unclear because embryos receive all the necessary nutrients from their mother plants^4^. During seed development, embryonic photosynthesis produces oxygen within the enclosed seed, and energy-rich compounds are required to fuel anabolic activities, thus contributing to whole-seed metabolism and carbon economy^5^. The role of photosynthesis in chloroembryos has also been associated with the rapid synthesis of adenosine triphosphate (ATP) and the reduced form of nicotinamide dinucleotide phosphate (NADPH)^6^.

During seed development, chloroplasts differentiate during the early phase of seed embryogenesis, after which the chloroplast dedifferentiation process leads to the presence of small nonphotosynthetic plastids in dry seeds^7^. Long-lived desiccation-tolerant seeds categorized as orthodox are assumed to contain dismantled thylakoids of chloroplasts^3^, but chloroplast dedifferentiation pathways have been documented only in the seeds of several species. Autophagic pathways of chloroplast degradation and/or dedifferentiation include whole chloroplast deposition into vacuoles, chloroplast stroma degradation into Rubisco-containing bodies (RCBs), further transport to vacuoles, and partial chloroplast or stroma sequestration to a senescence-associated vacuole (SAV) in the cytoplasm^8^.

Photosynthetic pigments usually become degraded during the maturation and drying of desiccation-tolerant tissues^9,10^. However, in some species, dried cells retain active chlorophyll^3^. For example, long-lived pea and bean seeds are classified as orthodox and have highly degraded photosystems^11,12^. Interestingly, short-lived cells maintain well-developed photosystems, such as those of chlorophyllous seeds and fern spores^13–15^.

*Acer platanoides* L. and *Acer pseudoplatanus* L. produce seeds that are tolerant and sensitive to desiccation, respectively. In this context, *A. platanoides* seeds are categorized as orthodox, whereas *A. pseudoplatanus* seeds are categorized as recalcitrant^16,17^. Little is known about the fate of chloroplasts in maturing and dry seeds of *A. platanoides*, whereas *A. pseudoplatanus* seeds were reported to contain well-defined plastids with an atypical granal structure and to possess the potential to fix CO_2_^18^. Distinct seed categories contribute to the differences in redox regulation of NAD(P) contents and redox states^19–21^, peroxiredoxin contents^22^, and methionine sulfoxide reductase B1 and B2 contents^23^ in the two *Acer* species. Chloroplasts are crucial for the redox state of plant cells^24,25^. Therefore, the fate of chlorophyll and chloroplasts in *Acer* seeds is hypothesized to play an important role in defining the physiological differences between *A. platanoides* and *A. pseudoplatanus* seeds. To test this hypothesis, we investigated the Chl *a* and Chl *b* contents and the autofluorescence signals of the chlorophyll and carotenoid contents in developing and dried *A. platanoides* and *A. pseudoplatanus* seeds.

## Results

### Chlorophyll *a* and *b* in *Acer platanoides* and *Acer pseudoplatanus* seeds

**Fig. 1:**
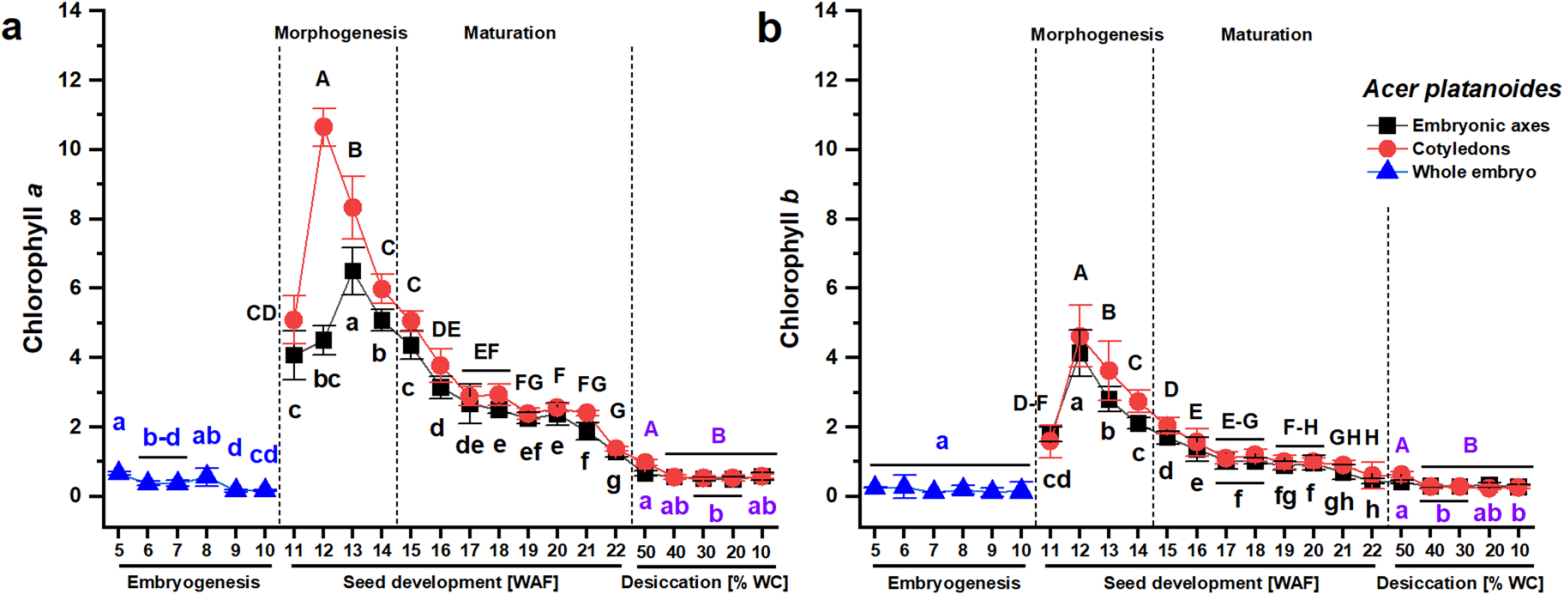
The contents of chlorophyll* a* (**A**) and chlorophyll *b* (**B**) in *Acer platanoides* seeds at the embryogenesis (whole embryo) stage, in separated embryonic axes and cotyledons at the seed development stage, consisting of seed morphogenesis and seed maturation, indicated with a dashed line, and in desiccated mature seeds. The data represent the means of at least six replicates ± the standard deviation. The content of chlorophylls is expressed in units of mg g^–1^ DW. Different letters indicate significant differences according to Tukey’s post hoc test performed separately for the following three stages: embryogenesis (blue font), seed development (black font), and seed desiccation (violet font). Capital letters refer to cotyledons, small letters refer to the whole embryo and embryonic axes. *WAF* weeks after flowering, *WC* water content.

The contents of Chl *a*, Chl *b*, and carotenoids (Car) were measured in developing *Acer platanoides* and *Acer pseudoplatanus* seeds 1) at the embryogenesis stage, 2) during the seed development (morphogenesis and maturation) stage, and 3) in dried seeds. The morphogenesis of *A. platanoides* seeds was slower, and beginning from the 11th week after flowering (WAF) to the collection date at the end of July, it was possible to physically detach the embryonic axes from the cotyledons because they were formed as two separate seed parts. At the embryogenesis stage, the data represent the content of photosynthetic pigments measured for the whole embryo (Fig. 1A, B). The contents of Chl *a* and *b* were significantly lower at the maturation stage than at the morphogenesis stage. The highest content of Chl *a* occurred a week earlier in the cotyledons (12th WAF) than in the embryonic axes (13th WAF) of *A. platanoides* and was twice as high in the cotyledons (Fig. 1A). The highest Chl *b* content occurred simultaneously at the 12th WAF in the embryonic axes and cotyledons (Fig. 1B). Seed desiccation caused a smooth decrease in chlorophylls in *A. platanoides* seeds, and their content was comparable to that observed during embryogenesis (5th–10th WAF). The Chl *a* content decreased five times (from 6.5 mg g^–1^ DW at 13th WAF to 1.3 mg g^–1^ DW at 22nd WAF) in the embryonic axes and eight times (from 10.6 mg g^–1^ DW at 12th WAF to 1.3 mg g^–1^ DW at 22nd WAF) in cotyledons during *A. platanoides* seed development (Fig. 1A), whereas the Chl *b* content decreased nine times in the embryonic axes (from 4.1 mg g^–1^ DW at 12th WAF to 0.45 mg g^–1^ DW at 22nd WAF) and eight times (from 4.6 mg g^–1^ DW at 12th WAF to 0.57 mg g^–1^ DW at 22nd WAF) in cotyledons (Fig. 1B).

**Fig. 2:**
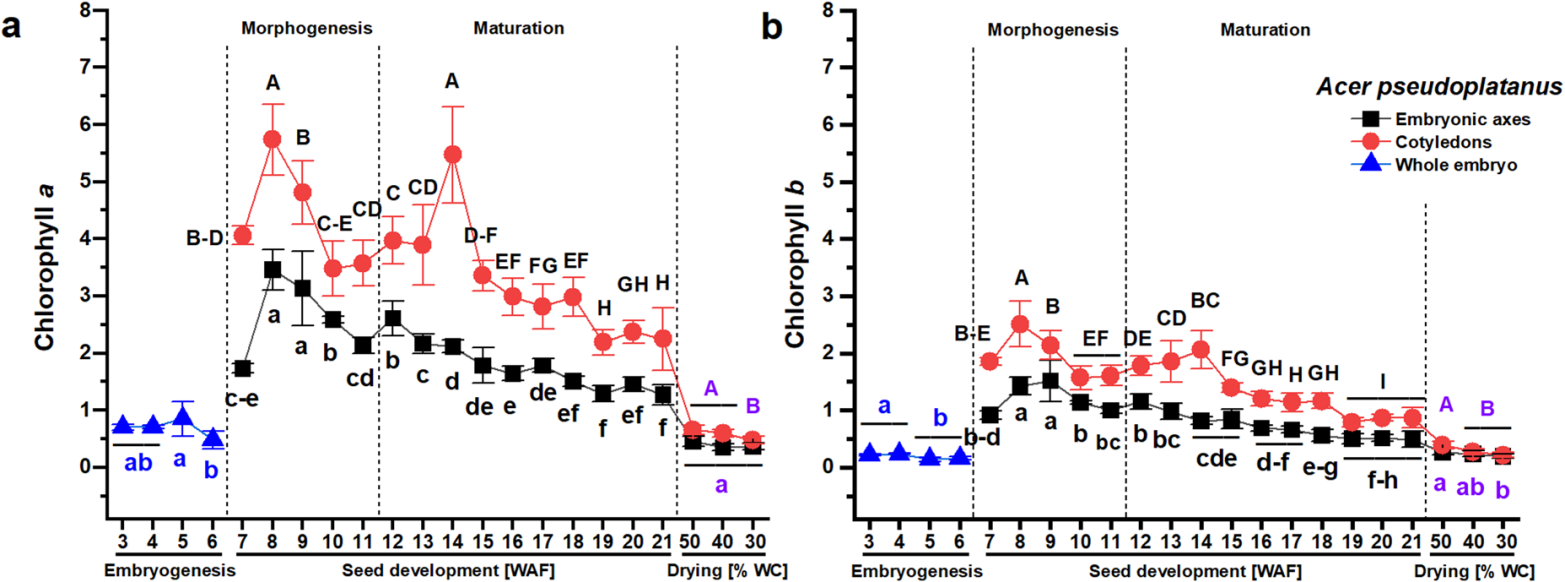
The contents of chlorophyll* a* (**A**) and chlorophyll *b* (**B**) in *Acer pseudoplatanus* seeds at the embryogenesis (whole embryo) stage, in separated embryonic axes and cotyledons at the seed development stage, consisting of seed morphogenesis and seed maturation, indicated with a dashed line, and in dehydrated mature seeds. The data represent the means of at least six replicates ± the standard deviation. The content of chlorophylls is expressed in units of mg g^–1^ DW. Different letters indicate significant differences according to Tukey’s post hoc test performed separately for the following three stages: embryogenesis (blue font), seed development (black font), and seed dehydration (violet font). Capital letters refer to cotyledons, small letters refer to the whole embryo and embryonic axes. *WAF* weeks after flowering, *WC* water content.

Morphogenesis was faster in *A. pseudoplatanus* seeds. At the 7th WAF, which was the collection date at the beginning of July, it was possible to physically separate the embryonic axes from the cotyledons; at earlier stages, the data represent the photosynthetic pigments measured for the entire embryo. The highest Chl *a* content occurred simultaneously at the 8th WAF in the embryonic axes and cotyledons, at the beginning of the maturation phase, and was nearly half as high in the cotyledons (Fig. 2A). Another peak in Chl *a* content was reported in the cotyledons at the 14th WAF. The highest Chl *b* content was reported one week earlier in the cotyledons (8th WAF) than in the embryonic axes (9th WAF) of *A. pseudoplatanus* (Fig. 2B). Seed dehydration from 58 to 50% WC under laboratory conditions caused a sharp decrease in the content of Chl *a*. Except for the seed drying phase, the content of both chlorophylls was greater in *A. pseudoplatanus* cotyledons than in the embryonic axes. In contrast, the embryonic axes and cotyledons of *A. platanoides* displayed relatively similar contents of chlorophylls. The decline in the chlorophyll content was similar in the embryonic axes and cotyledons of *A. pseudoplatanus* seeds and slightly differed between chlorophylls (2.5 for Chl *a* and three times for Chl *b*). More precisely, the Chl *a* content decreased in *A. pseudoplatanus* embryonic axes from 3.5 mg g^–1^ DW at 8th WAF to 1.3 mg g^–1^ DW at 21st WAF and from 5.7 mg g^–1^ DW at 8th WAF to 2.2 mg g^–1^ DW at 19th WAF in cotyledons (Fig. 2A). The Chl *b* content decreased in *A. pseudoplatanus* embryonic axes from 1.5 mg g^–1^ DW at 9th WAF to 0.5 mg g^–1^ DW at 19th WAF and from 2.5 mg g^–1^ DW at 8th WAF to 0.8 mg g^–1^ DW at 19th WAF in cotyledons (Fig. 2B).

The ratio of the Chl *a* to *b* content (Chl *a*/*b* ratio) significantly increased during the embryogenesis stage, when this ratio doubled both in the embryonic axes and cotyledons of *A. platanoides* (Table S1). From the establishment of the embryonic axes and cotyledons, the Chl *a*/*b* ratio decreased but again tended to increase as the seed maturation process progressed. Interestingly, the Chl a/b ratio was very high at the end of the embryogenesis of *A. pseudoplatanus* seeds, reaching values twice as high as those during the entire studied period. The Chl *a*/*b* ratio ranged between two and three, indicating a higher Chl *a* content in the seeds of both species. In the embryonic axes of *A. platanoides*, the Chl *a*/*b* ratio was relatively constant, but it clearly increased as the *A. pseudoplatanus* seeds matured. Drying of mature seeds caused a drastic decrease in the Chl a/b ratio, particularly in *A. platanoides* seeds. However, during progressive dehydration and desiccation, this ratio still increased.

**Fig. 3:**
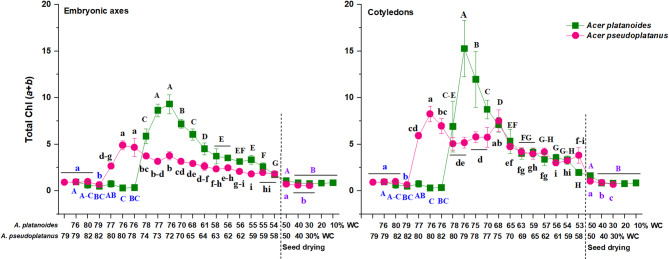
Total Chl (*a* + *b*) content reported at the embryogenesis, seed development, and drying stages in *Acer platanoides* and *Acer pseudoplatanus* seeds. The data represent the means of at least six replicates ± the standard deviation. Different letters indicate significant differences according to Tukey’s post hoc test performed separately for the following three stages: embryogenesis (blue font), seed development (black font), and seed drying (violet font). Capital letters refer to *A. platanoides* seeds, small letters refer to *A. pseudoplatanus*. *WC* water content.

The total Chl (*a* + *b*) content increased during morphogenesis but then decreased during maturation (Fig. 3). The total Chl content decreased fivefold in the embryonic axes of *A. platanoides* (from 9.2 mg g^–1^ DW at 13th WAF to 1.7 mg g^–1^ DW at 22nd WAF) and twofold in *A. pseudoplatanus* (from 4.9 mg g^–1^ DW at 8th WAF to 1.8 mg g^–1^ DW at 20th WAF), whereas in the cotyledons, it decreased sevenfold in *A. platanoides* (from 15.3 mg g^–1^ DW at 12th WAF to 2.0 mg g^–1^ DW at 22nd WAF) and twofold in *A. pseudoplatanus* (from 8.2 mg g^–1^ DW at 8th WAF to 3.0 mg g^–1^ DW at 19th WAF). The total chlorophyll content was the highest in *A. platanoides* seeds in the embryonic axes during the entire maturation stage and in the cotyledons during the initial phase of maturation. In mature and dried seeds, the total content of chlorophyll was equal in both maple species.

**Fig. 4:**
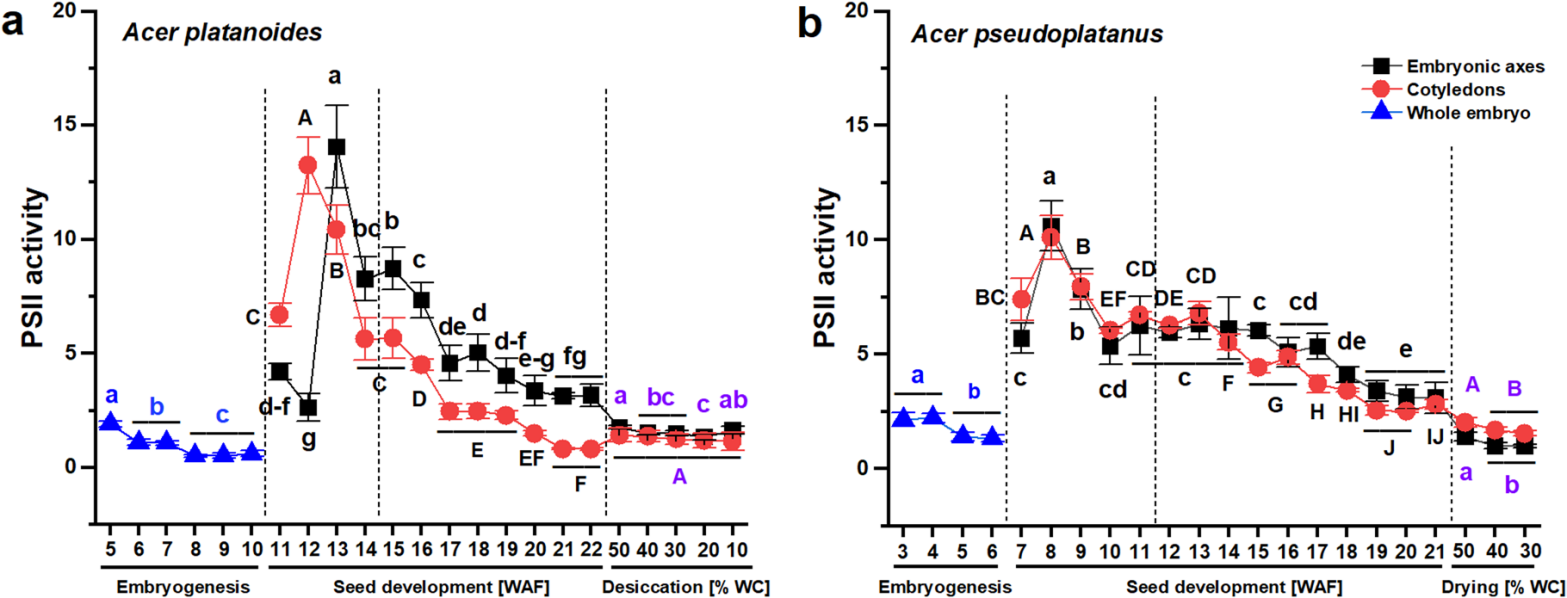
The activity of photosystem II (PSII) at the embryogenesis (whole embryo) stage, in separated embryonic axes and cotyledons at the seed development stage, consisting of seed morphogenesis and seed maturation, indicated with a dashed line, and in dried mature seeds of *Acer platanoides* (**A**) and *Acer pseudoplatanus* (**B**). The data represent the means of at least six replicates ± the standard deviation. The activity of PSII is expressed in units of RFU × 10^–6^ g^–1^ DW. Different letters indicate significant differences according to Tukey’s post hoc test performed separately for the following three stages: embryogenesis (blue font), seed development (black font), and seed drying (violet font). Capital letters refer to cotyledons, small letters refer to the whole embryo and embryonic axes. *WAF* weeks after flowering, *WC* water content, *RFU* relative fluorescence unit.

The activity of photosystem II (PSII), as measured by fluorescence, might reflect photosynthetic activity. The activity of PSII was greater in *A. pseudoplatanus* seeds than in *A. platanoides* seeds at specific developmental stages (Fig. 4). More precisely, higher activity of PSII was reported in *A. pseudoplatanus* embryonic axes at the 5th–12th and 17th–18th WAF and in the cotyledons at the 6th–10th and 11th–21st WAF as well as during drying from 50 to 30% WC compared with that in *A. platanoides* seeds (Table S2). The activity of PSII was reduced four times (from 14 RFU g^–1^ DW at 13th WAF to 3.2 RFU g^–1^ DW at 22nd WAF) in the embryonic axes and sixteen times (from 13.2 RFU g^–1^ DW at 12th WAF to 0.81 RFU g^–1^ DW at 22nd WAF) in cotyledons during *A. platanoides* seed development (Fig. 4A), whereas in the embryonic axes of *A. pseudoplatanus* the activity of PSII was reduced three times (from 10.6 RFU g^–1^ DW at 8th WAF to 3.1 RFU g^–1^ DW at 21st WAF) and four times (from 10.1 RFU g^–1^ DW at 8th WAF to 2.5 RFU g^–1^ DW at 20th WAF) in cotyledons (Fig. 4B).

### Carotenoids in *Acer platanoides* and *Acer pseudoplatanus* seeds

**Fig. 5:**
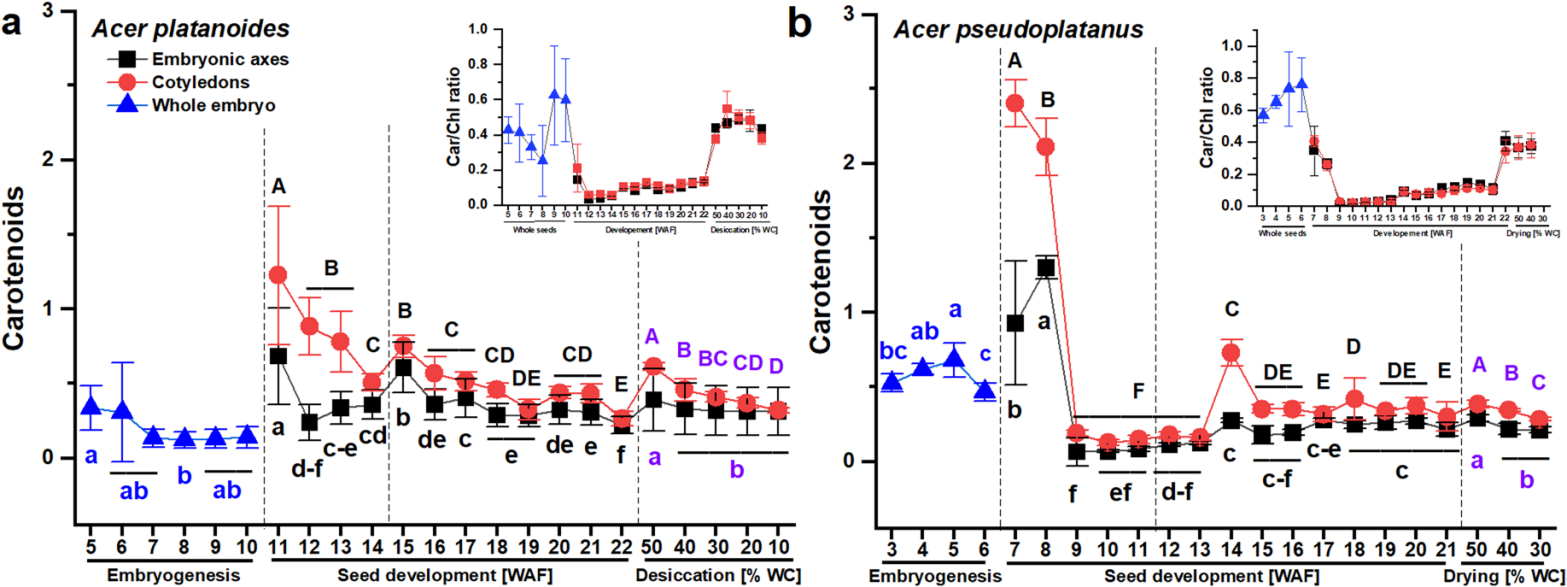
The content of carotenoids (Car) reported at the embryogenesis (whole embryo) stage, in separated embryonic axes and cotyledons at the seed development stage, consisting of seed morphogenesis and seed maturation, indicated with a dashed line, and in dried mature seeds of *Acer platanoides* (**A**) and *Acer pseudoplatanus* (**B**), accompanied by the ratio of the Car to chlorophyll (Chl) content presented in the inset plot (for more detailed data, see Table S2). The data represent the means of at least six replicates ± the standard deviation. The content of carotenoids is expressed in units of mg g^–1^ DW. Different letters indicate significant differences according to Tukey’s post hoc test performed separately for the following three stages: embryogenesis (blue font), seed development (black font), and seed drying (violet font). Capital letters refer to cotyledons, small letters refer to the whole embryo and embryonic axes. *WAF* weeks after flowering, *WC* water content.

The Car content was much greater in *A. pseudoplatanus* seeds at the embryogenesis stage and tended to increase in the embryonic axes at the beginning of seed morphogenesis, which was not observed in *A. platanoides* seeds (Fig. 5). Considering the antioxidant function of Car, the Car/Chl ratio, which reflects the resistance of a seed to stress conditions, was examined (Fig. 5, Table S3). The embryogenesis stage was characterized by a significant contribution of Car to the pigment pool, whereas at the maturation stage, the amount of chlorophyll dominated because the Car/Chl ratio did not exceed 0.15 in either species and tended to increase until the seeds reached maturity (Table S3). Considering the different initial values of both pigments, this index increased twofold in *A. platanoides* seeds at the maturation stage, whereas in *A. pseudoplatanus* seeds, sixfold and fourfold increases were detected in the embryonic axes and cotyledons, respectively. Despite the increase in the Car/Chl ratio during maturation, its final value was similar in the embryonic axes of both species but remained higher in *A. pseudoplatanus* cotyledons. The Car/Chl ratio increased again in the dried seeds.

### Visualization of the chlorophyll in *Acer platanoides* and *Acer pseudoplatanus* seeds

**Fig. 6:**
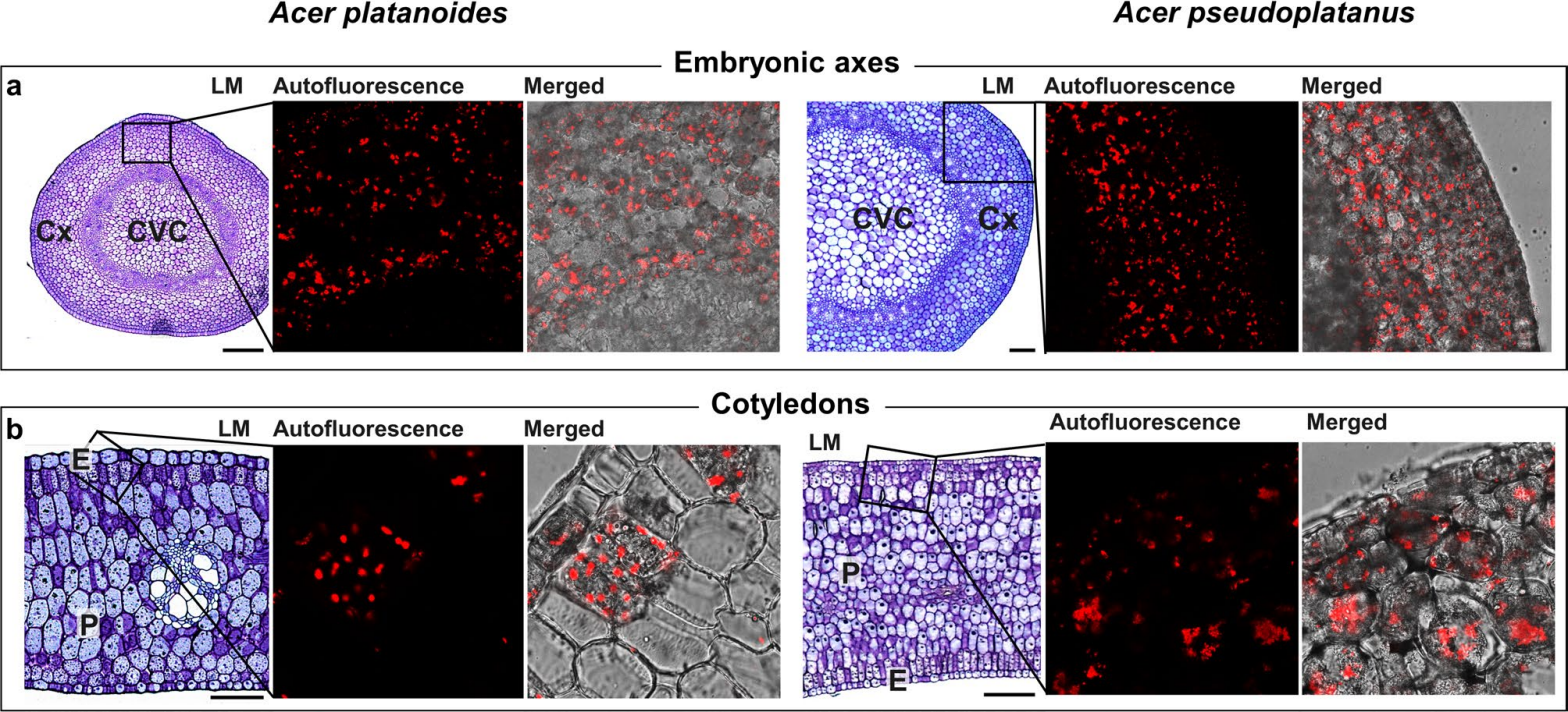
The structure of the embryonic axes (**A**) and cotyledons (**B**) of *Acer platanoides* and *Acer pseudoplatanus* seeds, and chlorophyll autofluorescence in these tissues detected using a confocal microscope. Bars – 100 µm. *Cx* cortex precursor cells, *CVC* central vascular cylinder cells, *P* parenchyma cells, *E* epidermis, *LM* light microscopy.

The cortex precursor cells (Cx) from embryonic axes and parenchyma cells (P) located directly below the epidermis (E) in the cotyledons of mature seeds from *A. platanoides* and *A. pseudoplatanus* were selected for microscopic observation (Fig. 6). Chlorophyll autofluorescence was observed in the embryonic axes (Fig. 6A) and cotyledons (Fig. 6B). For both *Acer* species, the autofluorescence signal was irregular and somewhat diffuse in the embryonic axes. The same irregular and diffuse signal was present in *A. pseudoplatanus* cotyledons, whereas *A. platanoides* cotyledons also displayed the second pattern of autofluorescence, in which the signal was spherical and more compact.

## Discussion

*Acer platanoides* trees flower three weeks earlier than *Acer pseudoplatanus* trees; however, *A. pseudoplatanus* seeds develop more rapidly, and eventually, the seeds of both species are fully mature in October^26^. In this study, embryonic axes and cotyledons could be distinguished as separate embryo parts in July; however, they could be distinguished three weeks earlier in *A. pseudoplatanus* than in *A. platanoides*. At the embryogenesis stage, the whole embryo was the only tissue available to analyze. Schemes visualizing the structure of *A. platanoides* and *A. pseudoplatanus* seeds^26^ and photographic documentation of *A. platanoides* development^27^ demonstrated that beginning from the 12th WAF, embryonic axes and cotyledons develop in *A. platanoides* seeds^26,27^, whereas in *A. pseudoplatanus* seeds, this phenomenon occurs at the 7th WAF^26^. In our study, embryonic axes and cotyledons formed at the 8th WAF in *A. pseudoplatanus* and the 11th WAF in *A. platanoides*. This can be explained by the phenomenon of earlier flowering in warmer environments by approximately 3 days per 1 °C increase in spring-onset average temperatures^28^. For example, the average air temperature in Poland in 2022–24 was 10.0 °C, approximately 1.3 °C higher than the average temperature from 1991–2020 (IMGW).

Measurements of chlorophyll fluorescence indicate PSII activity and photochemical efficiency^29^. In this analysis, the peak fluorescence of PSII (Fig. 4) coincided with the total chlorophyll content (Fig. 3), suggesting that photosynthesis might occur in developing *Acer* seeds and that its efficiency was greater in *A. pseudoplatanus* seeds at the initial and final developmental stages, and in dried seeds. Chloroembryos have been confirmed to carry out photosynthesis. For example, developing *Arabidopsis thaliana* embryos are green and photosynthetically active^7^. The photosynthetic activity in embryos has been reported to appear at the globular stage, coinciding with plastid differentiation into mature chloroplasts and the accumulation of chlorophyll^30^. Starch granules reported in *A. pseudoplatanus* chloroplasts confirm the photosynthetic activity in *A. pseudoplatanus* seeds^18^.

Chloroplast-containing cells are specifically distributed at the globular, heart, torpedo, and walking-stick stages of Arabidopsis embryogenesis^30^. Chloroplast differentiation occurs naturally during leaf senescence^31,32^. Dismantlement of chloroplasts into nonphotosynthetic eoplasts was reported in developing maize seeds and attributed to a factor required for seed longevity^33^. In this study, the round and denser chlorophyll autofluorescence signal reported in *A. platanoides* cotyledons (Fig. 6B) suggests the possible dedifferentiation of some chloroplasts from the chloroplast pool. For example, the most characteristic morphological features of chloroplast degeneration, hence the transition of chloroplasts to eoplasts, are volume alterations, and the transition from ellipsoid to circular morphology, is combined with a deep reorganization of internal membranes. To verify whether chloroplasts in *A. platanoides* seeds undergo differentiation, degradation, or autophagy^34^, transmission electron microscopy observations are needed. However, volume alterations were clearly reported in *A. platanoides* cotyledons (Fig. 6). Seeds characterized by a green color are termed chloroembryos, whereas chlorophyllous cells refer to cells with intact chloroplasts. In this context, *A. platanoides* is hypothesized to produce chloroembryos, whereas *A. pseudoplatanus* seeds might be considered to contain chlorophyllous cells on the basis of the findings of Pinfield and colleagues^18^.

Our recent omics findings^35^ demonstrated that seed imbibition causes the activation of different genes and the synthesis of different proteins and metabolites related to the reactivation of organelles in *A. platanoides* compared with those in *A. pseudoplatanus* seeds, suggesting that some pools of chloroplasts in *A. platanoides* seeds might have undergone dedifferentiation. The dedifferentiation of chloroplasts is accompanied by seed degreening because of the loss of chlorophyll^13^. In this context, the changes in chloroplast structure might have appeared beginning at the 13th WAF in *A. platanoides* seeds and at the 16th WAF in *A. pseudoplatanus* (Figs. 1 and 2) when the loss of chlorophylls was evident. Chlorophyll degradation is regulated hormonally, mainly by ABA, and genetically via SGR proteins encoded by *STAY GREEN* genes, the pheophorbide a oxygenase (PAO) pathway, and Chl *b* reductases^36^. Sometimes, however, chlorophyll degradation does not occur, and dried cells retain active chlorophyll^3^. For example, *Salix* *nigra* seeds maintain their chlorophyll, chloroplasts, and thylakoids^14^, and although they are classified as orthodox seeds^37^, they remain viable for only a few weeks when stored at room temperature^38^. The cotyledons of pea seeds, which are still green and categorized as long-lived orthodox^39^, contain 10% of the chlorophyll levels detected in *Salix nigra* cotyledons^40^. In general, the chlorophyll content is inversely related to seed quality^41^. Mature *A. platanoides* and *A. pseudoplatanus* seeds displayed similar total chlorophyll contents in their embryonic axes, and slightly lower levels were found in *A. platanoides* than in *A. pseudoplatanus* cotyledons (Figs. 1–3). This difference is caused by the higher levels of Chl *a* in *A. pseudoplatanus* cotyledons than in *A. platanoides* cotyledons (Figs. 1 and 2). This finding suggests that the initial quality of mature *Acer* seeds is similar. The photosynthesis of selected seed tissues (cotyledons, embryonic axes, and fruit pericarps) might differ because chlorophyll is restricted predominantly to the cotyledons^5^. Considering that NADPH is rapidly synthesized during photosynthesis^6^, the constant decrease in NADPH content observed only in the cotyledons of developing *A. platanoides* seeds^42^ might indicate a gradual decline in photosynthetic activity. Moreover, the NADPH content fluctuated without any decreasing trend throughout seed development in *A. pseudoplatanus* cotyledons^42^. The continuous decrease in NADPH content reported in developing *A. platanoides* cotyledons could be related to gradual metabolic shutdown mechanisms, which are characteristic of orthodox-category seeds^43^; however, a different pattern of changes in NADPH content was observed in *A. platanoides* embryonic axes, and a peak in NADPH was observed at the 19th WAF, which coincided with the acquisition of desiccation tolerance, a phenomenon recognized to occur at the 18th WAF^44^. We suggest that the decreasing NADPH content in *A. platanoides* cotyledons possibly reflects declining photosynthetic activity because the unique condensed and spherical autofluorescence signal of chlorophyll was also reported uniquely in *A. platanoides* cotyledons (Fig. 6). Additionally, the content of NADPH in the embryonic axes of developing *A. pseudoplatanus* seeds was the highest at the beginning and final stages of seed development^42^, which coincided with the elevated PSII activity reported at the corresponding stages (Fig. 4). Such a phenomenon was not observed in *A. pseudoplatanus* cotyledons because embryonic axes and cotyledons function differently in recalcitrant seeds, even upon storage. In contrast to cotyledons, embryonic axes can alter their metabolism via the active synthesis of new proteins that act on metabolism^45,46^.

Chlorophyll retention affects seed longevity^41^. For example, Arabidopsis mutants that retain 10 times more chlorophyll in their seeds exhibit a decreased germination capacity during storage^41^, whereas seeds with lower amounts of chlorophyll display high germination rates^47^. Chlorophyll degradation is induced by abscisic acid (ABA) and occurs via chlorophyll reductases (i.e., by chlorophyll I reductase, which converts Chl *b* to Chl *a*) and SGR proteins, which degrade Chl *a* to Phein *a* via the extraction of Mg^2+^^36,41,48^. In this study, the decrease in Chl *b* did not increase the content of Chl *a* (Figs. 1 and 2), indicating the putative action of SGR proteins in the full chlorophyll degradation pathway.

Recalcitrant seeds of the *Acer* genus, including *A. pseudoplatanus*, can escape this category and become more desiccation-tolerant^49^. High seed mass is an attribute of recalcitrance^50^. The mass of the analyzed mature *A. pseudoplatanus* seeds was 67 mg on average, which fit the normal values characteristic of Poland^51^. In this context, *A. pseudoplatanus* recalcitrance was not shifted toward a more desiccation-tolerant phenotype. Importantly, degradation of chlorophyll was observed in both species (Figs. 1–3) and was not restricted to a single seed category. However, the reduction in chlorophylls was greater in *A. platanoides* than in *A. pseudoplatanus* seeds. Moreover, *A. pseudoplatanus* seeds, even if successful in terms of chlorophyll degradation (Figs. 1–3), seem to fail at chloroplast dedifferentiation^18^.

In plants, Cars are light-harvesting pigments that are essential components of photosystems and display protective activity^52^. In seeds, Cars are crucial for ABA synthesis, the acquisition of seed dormancy, and the activity of antioxidant systems that prevent age-related deterioration of membrane structures^53,54^. Car biosynthesis and storage occur mainly in plastids, including in dark-grown tissues^52^. The highest concentration of Car was detected in *Acer* seeds before the peak chlorophyll synthesis occurred (Fig. 5). This can be explained by the fact that Car, together with the chlorophyll precursor, accumulate together in the prolamellar body (PLB) of dark-grown tissues, which contain the membranous structure required for chloroplast formation^55^. The elevated levels of Car reported during the drying of seeds detached from the mother tree might suggest a protective effect in response to water loss because the *A. platanoides* seed tissues contained higher Car levels than those of the recalcitrant-seeded species, *A. pseudoplatanus* (Fig. 5). The Car/Chl ratio was suggested to reflect the resistance of a seed to stress conditions^56^. The higher Car/Chl ratio reported at embryogenesis and during drying in *A. pseudoplatanus* seeds (Fig. 5, Table S3) might indicate the involvement of Car in the protection of recalcitrant *A. pseudoplatanus* seeds; however, during the maturation stage, the seeds of both *Acer* species displayed similar Car/Chl ratios; therefore, this ratio does not predispose any species to be more resistant to stress factors at this stage.

## Conclusion

This study provides evidence for chlorophyll degradation in maturing *Acer platanoides* and *Acer pseudoplatanus* seeds. Greater degradation was evident for *A. platanoides* seeds and is characteristic of orthodox seeds. Only *A. platanoides* seeds exhibited symptoms of structural changes in chloroplasts in mature seeds. Both of the above phenomena possibly contribute to seed longevity in *A. platanoides* seeds. In contrast, although *A. pseudoplatanus* seeds degrade chlorophyll to some extent, chloroplasts possibly remain unchanged, and more detailed microscopic analyses will resolve this issue. Nevertheless, less pronounced reductions in the content of photosynthetic pigments and less drastic downregulation of photosynthetic activity in *A. pseudoplatanus* potentially reflect different adaptive strategies. These strategies are implemented in the seed development program, which determines the reduced longevity of recalcitrant seeds.

## Materials and methods

### Seed material

Developing *Acer platanoides* and *Acer pseudoplatanus* seeds were collected from single trees in Kórnik (2°24′37′′N, 17°09′515′′E) in three consecutive harvesting years, 2022–2024. Trees of both species grow less than 50 m apart in podzolic soil with an atmospheric humidity regime type, pH 6.2–7.7 corresponding to 20–150 cm of depth, and at identical climatic conditions characteristic of Western Poland. Their crowns are equally exposed to sunlight. The collection started weekly in July, and whole embryos were analyzed at the 5th–10th WAF in *A. platanoides* and the 3rd–6th WAF in *A. pseudoplatanus*, reflecting the embryogenesis stage from the preglobular stage to the torpedo stage^57^. Afterward, the separation of embryonic axes and cotyledons was possible because they were formed as two distinct seed tissues. Therefore, embryonic axes and cotyledons were used for analyses at the seed development stages in *A. platanoides* (11th–22nd WAF) and *A. pseudoplatanus* (7th–21st WAF). Seed development includes the morphogenesis and maturation phases^57^. Morphogenesis in *A. platanoides* ranged from the 11th–14th WAF^27^, followed by the maturation stage, which ranged from the 15th to 22nd WAF. According to the defined seed development stages^57^, morphogenesis in *A. pseudoplatanus* is assumed to range from the 7th–11th WAF and maturation ranged from the 12th–21st WAF. Each collection time, the seeds were excised from the tree and immediately prepared for freezing at –80 °C by removing the samara and seed testa. Mature seeds were dried at room temperature (25 °C). *A. platanoides* seeds were gradually desiccated, and seed samples were prepared with 50%, 40%, 30%, 20%, and 10% WC. *Acer pseudoplatanus* desiccation-sensitive seeds were gradually dehydrated, and seed samples were prepared with 50%, 40%, and 30% WC.

The data represent means from at least six biological replicates per available seed collection time across two harvesting years, or more replicates per time point across three harvesting years. For each biological replicate, we performed three technical replicates to measure absorbance and fluorescence on an Infinite M200 PRO plate reader (Tecan, Männedorf, Switzerland).

### Determination of photosynthetic pigments

The pigments involved in photosynthesis (chlorophylls and carotenoids) were isolated in a 90:10 (v/v) acetone:Milli-Q H_2_O solvent system. The concentration of chlorophylls was calculated according to the method described by Ritchie^58^ and on the basis of the equations below, considering the wavelengths λ = 647 nm and λ = 663 as the maximum of Chl *b* and Chl *a* absorption, respectively:

$${\mathrm{Chl}}~a = - {1}.{7858} \times {\mathrm{A}}_{{{647}}} + {11}.{8668} \times {\mathrm{A}}_{{{663}}}$$

$${\mathrm{Chl}}~b = { 18}.{9775} \times {\mathrm{A}}_{{{647}}} - {4}.{895} \times {\mathrm{A}}_{{{663}}}$$

$${\text{Total chlorophyll }} = {\mathrm{Chl}}~a + {\mathrm{Chl}}~b$$

The contents of Chl *a* and Chl *b* were calculated on the basis of the dry weight of the seed samples and expressed in units of mg g^–1^ DW. Calculations include a correction factor considering 1.0 cm^3^ (1 mL) of sample and a 1 cm path length used for standard cuvette-based absorbance measurements and the establishment of the equations to transform our data obtained from the plate reader.

The content of Car was calculated in the same units as that used for chlorophylls and was based on the following Eq.^59^:

$${\mathrm{Car}}\, = \,\left( {{1}000\, \times \,{\mathrm{A}}_{{{47}0}} {-}{3}.{27}\, \times \,{\mathrm{A}}_{{{663}}} - {1}0{4}\, \times \,{\mathrm{A}}_{{{647}}} } \right)/{198}$$

### PSII activity

Photosystem II activity was measured on the basis of the chlorophyll fluorescence capacity. The fluorescence (λex = 435/λem = 676 nm) was measured using an Infinite M200 PRO (Tecan, Männedorf, Switzerland) plate reader and Magellan software. The results are shown in relative fluorescence units (RFU) 10^−6^ per gram of DW.

### Light microscopy

Mature embryonic axes and cotyledons were fixed and embedded in Technovit resin (Heraeus Kulzer, Wehrheim, Germany) according to the protocol described by Wojciechowska et al.^59^. The cross-sections were cut with a Leica RM2265 fully automated rotary microtome (Leica-Reichert, Bensheim, Germany) at a thickness of 10 μm. These cross-sections were stained with 0.5% (m/v) toluidine blue (pH 6.8) and examined under a light microscope (Axioscope A1; Carl Zeiss, Jena, Germany).

### Autofluorescence of chlorophyll

Cotyledons were fragmented into 5 mm × 5 mm pieces, and the whole embryonic axes were fixed in a solution containing 2% glutaraldehyde (Polysciences, Warrington, FL, USA) and 2% (v/v) formaldehyde (Polysciences, Warrington, FL, USA) for 12 h at 4 °C. Following fixation, the material was rinsed three times in 0.1 M phosphate-buffered saline (PBS) and sectioned into 30 µm slices using a Leica VT 1200S vibratome (Leica Biosystems, Nussloch, Germany). Confocal imaging was performed with a Leica Stellaris DMi8 microscope (Leica Biosystems, Nussloch, Germany) equipped with a white light laser (WLL) set to 453 nm for optimal excitation of Chl *b* autofluorescence. Emission was measured in the red spectral range (680–700 nm) corresponding to the fluorescence maximum of chlorophyll. In addition, transmitted light images were acquired to visualize tissue morphology in parallel with fluorescence channels.

### Statistical analysis

The obtained data were subjected to analysis of variance (ANOVA), and the significance of differences was determined using Tukey’s test, with a significance level of *p* ≤ 0.05. The statistics were performed separately for each of the three stages: embryogenesis, seed development, and seed drying. The statistical significance of the difference between the means of two groups was determined using a t test, with a significance level of *p* ≤ 0.05. Statistical analyses were performed using JMP® Pro 18.

## Supplementary Information

Supplementary Information.

## Data Availability

All data supporting the results are archived in the figshare repository with the following 10.6084/m9.figshare.30509939, and will be published on the day of the acceptance of this manuscript.
