# Supplementary material for: Photosynthetic pigments in developing seeds of Acer platanoides and Acer pseudoplatanus
Source: Sci Rep. 2026 Mar 21;16:14443. doi: 10.1038/s41598-026-44414-7 (PMC13149657; doi:10.1038/s41598-026-44414-7)
Supplement: Supplementary file 1 — Supplementary Information. [file 41598_2026_44414_MOESM1_ESM.pdf]

Photosynthetic pigments in developing seeds of *Acer platanoides* and *Acer pseudoplatanus*

Amir Mohammad Mokhtari, Natalia Wojciechowska, Andrzej Kowalski, Ewa Marzena Kalemba

Table S1. The ratio of chlorophyll *a* to chlorophyll *b* reported in whole developing seeds, separated embryonic axes (E. axes) and cotyledons of developing seeds, and dried mature seeds of *Acer platanoides* and *Acer pseudoplatanus*. Data are the means of at least six replicates ± standard deviation. Different letters indicate significant differences according to Tukey's post hoc test performed separately in the embryogenesis, seed development, and seed drying stages. WAF, weeks after flowering. Color used to fill table cells: light yellow refers to the embryogenesis stage; green colors refer to the seed developmental stage, and within this stage, lighter green refers to the morphogenesis stage, and darker green refers to the maturation stage; pink refers to the seed drying (dehydration or desiccation) stage.

Stage	*Acer platanoides*		*Acer pseudoplatanus*	
	**E. axes**	**Cotyledons**	**E. axes**	**Cotyledons**
**3 WAF**			3.33 ± 0.12 b	
**4 WAF**			2.96 ± 0.12 b	
**5 WAF**	2.64 ± 0.20 bc		7.69 ± 0.97 a	
**6 WAF**	2.32 ± 0.32 c		2.55 ± 0.06 b	
**7 WAF**	2.74 ± 0.21 b		2.11 ± 0.17 ef	2.24 ± 0.11 g
**8 WAF**	3.00 ± 0.37 b		2.38 ± 0.05 b-f	2.30 ± 0.18 fg
**9 WAF**	3.65 ± 0.11 a		2.11 ± 0.33 f	2.25 ± 0.03 g
**10 WAF**	2.29 ± 0.07 c		2.28 ± 0.04 d-f	2.21 ± 0.06 g
**11 WAF**	1.19 ± 0.18 d	3.39 ± 0.55 a	2.14 ± 0.02 ef	2.22 ± 0.04 g
**12 WAF**	2.52 ± 0.07 a-c	2.31 ± 0.05 de	2.26 ± 0.02 ef	2.22 ± 0.05 g
**13 WAF**	2.32 ± 0.08 c	2.29 ± 0.07 de	2.23 ± 0.18 ef	2.10 ± 0.07 h
**14 WAF**	2.41 ± 0.08f c	2.18 ± 0.03 e	2.59 ± 0.12 a-d	2.66 ± 0.05 bc
**15 WAF**	2.58 ± 0.08 bc	2.48 ± 0.06 b-d	2.12 ± 0.17 e-f	2.41 ± 0.07 e-f
**16 WAF**	2.38 ± 0.31 c	2.43 ± 0.06 cd	2.39 ± 0.11 c-e	2.48 ± 0.04 d-e
**17 WAF**	2.59 ± 0.18 bc	2.61 ± 0.04 bc	2.70 ± 0.08 ab	2.48 ± 0.14 d-e
**18 WAF**	2.47 ± 0.21 c	2.49 ± 0.12 b-d	2.72 ± 0.26 ab	2.57 ± 0.07 cd
**19 WA.F**	2.57 ± 0.19 bc	2.41 ± 0.05 cd	2.61 ± 0.33 a-c	2.76 ± 0.08 a
**20 WAF**	2.50 ± 0.39 bc	2.58 ± 0.07 bc	2.85 ± 0.13 a	2.73 ± 0.09 ab
**21 WAF**	2.76 ± 0.41 ab	2.66 ± 0.10 b	2.66 ± 0.35 a-c	2.57 ± 0.12 cd
**22 WAF**	2.91 ± 0.25 a	2.60 ± 0.55 bc		
**50% WC**	1.37 ± 0.11 bc	1.35 ± 0.11 b	1.52 ± 0.22 a	1.49 ± 0.15 b
**40% WC**	1.63 ± 0.15 b	1.77 ± 0.28 a	1.35 ± 0.33 a	1.60 ± 0.21 ab
**30% WC**	1.58 ± 0.19 b	1.73 ± 0.16 a	1.75 ± 0.29 a	1.79 ± 0.12 a
**20% WC**	1.30 ± 0.13 c	1.95 ± 0.24 a		
**10% WC**	1.91 ± 0.19 a	1.89 ± 0.17 a		

Table S2. Testing of the statistical significance of the difference between the means of PSII activity between two species *Acer platanoides* and *Acer pseudoplatanus*. The significance was tested using the T test at $p < 0.05$. Font color code: **blue** – significantly higher in sycamore, **red** – significantly higher in Norway maple, **black** – nonsignificant. Color used to fill table cells: light yellow refers to the embryogenesis stage; green colors refer to the seed developmental stage, and within this stage, lighter green refers to the morphogenesis stage, and darker green refers to the maturation stage; pink refers to the seed dehydration stage. * - data from the whole embryos

**Developmental stage**	***Acer platanoides*****vs *Acer pseudoplatanus***	
	**Embryonic axes**	**Cotyledons**
5 WAF	p = 0.00011*	p = 0.00011*
6 WAF	p = 0.012849*	p = 0. 012849
7 WAF	p < 0.00001*	p < 0.00001
8 WAF	p < 0.00001*	p < 0.00001
9 WAF	p < 0.00001*	p < 0.00001
10 WAF	p < 0.00001*	p < 0.00001
11 WAF	p = 0.001184	p = 0.434602
12 WAF	p < 0.00001	p < 0.00001
13 WAF	p < 0.00001	p < 0.00001
14 WAF	p = 0.000756	p = 0.392283
15 WAF	p < 0.00001	p = 0.000375
16 WAF	p < 0.00001	p = 0.009463
17 WAF	p = 0.13483	p < 0.00001
18 WAF	p = 0.00315	p < 0.00001
19 WAF	p = 0.024815	p = 0.007915
20 WAF	p = 0.214305	p < 0.00001
21 WAF	p = 0.436535	p < 0.00001
22 WAF		
50 % WC	p = 0.000873	p = 0.000874
40 % WC	p = 0.000013	p = 0.005431
30 % WC	p < 0.00001	p = 0.008024

Table S3. The ratio of carotenoids to chlorophyll calculated for the whole developing seeds, separated embryonic axes (E. axes) and cotyledons of developing seeds, and dried mature seeds of *Acer platanoides* and *Acer pseudoplatanus* . Data are the means of at least six replicates ± standard deviation. Different letters indicate significant differences according to Tukey's post hoc test. Color used to fill table cells: light yellow refers to the embryogenesis stage; green colors refer to the seed developmental stage, and within this stage, lighter green refers to the morphogenesis stage, and darker green refers to the maturation stage; pink refers to the seed drying (dehydration or desiccation) stage. WAF, weeks after flowering

	Acer platanoides		*Acer pseudoplatanus*	
	**E. axes**	**Cotyledons**	**E. axes**	**Cotyledons**
**3 WAF**			0.57 ± 0.04 a	
**4 WAF**			0.65 ± 0.04 a	
**5 WAF**	0.43 ± 0.07 ab		0.74 ± 0.23 a	
**6 WAF**	0.41 ± 0.16 ab		0.76 ± 0.16 a	
**7 WAF**	0.33 ± 0.07 ab		0.34 ± 0.16 a	0.40 ± 0.03 a
**8 WAF**	0.25 ± 0.20 b		0.27 ± 0.03 b	0.26 ± 0.03 b
**9 WAF**	0.62 ± 0.28 a		0.008 ± 0.04 i	0.03 ± 0.002 h
**10 WAF**	0.59 ± 0.23 a		0.02 ± 0.001 hi	0.02 ± 0.001 h
**11 WAF**	0.14 ± 0.01 a	0.21 ± 0.13 a	0.03 ± 0.004 hi	0.03 ± 0.003 h
**12 WAF**	0.03 ± 0.003 f	0.06 ± 0.003 e	0.03 ± 0.001 hi	0.03 ± 0.002 h
**13 WAF**	0.04 ± 0.003 f	0.06 ± 0.003 e	0.04 ± 0.005 g-i	0.03 ± 0.005 h
**14 WAF**	0.05 ± 0.004 f	0.06 ± 0.003 e	0.09 ± 0.008 d-f	0.09 ± 0.004 d-f
**15 WAF**	0.10 ± 0.008 cd	0.11 ± 0.005 cd	0.07 ± 0.02 f-h	0.07 ± 0.003 g
**16 WAF**	0.08 ± 0.02 e	0.11 ± 0.006 cd	0.08 ± 0.006 e-g	0.09 ± 0.003 e-g
**17 WAF**	0.11 ± 0.007 bc	0.13 ± 0.007 bc	0.11 ± 0.005 c-e	0.08 ± 0.008 fg
**18 WAF**	0.09 ± 0.01 e	0.11 ± 0.006 cd	0.12 ± 0.02 c-e	0.10 ± 0.02 cd
**19 WA.F**	0.09 ± 0.01 de	0.09 ± 0.004 d	0.15 ± 0.02 c	0.12 ± 0.005 c
**20 WAF**	0.10 ± 0.02 cd	0.12 ± 0.005 bc	0.14 ± 0.005 cd	0.11 ± 0.007 c
**21 WAF**	0.13 ± 0.03 ab	0.13 ± 0.007 bc	0.11 ± 0.03 c-f	0.10 ± 0.005 c-e
**22 WAF**	0.14 ± 0.008 a	0.14 ± 0.03 b		
**50% WC**	0.44 ± 0.03 a	0.38 ± 0.02 b	0.41 ± 0.06 a	0.34 ± 0.07 a
**40% WC**	0.44 ± 0.03 a	0.55 ± 0.10 a	0.37 ± 0.06 a	0.37 ± 0.07 a
**30% WC**	0.47 ± 0.03 a	0.50 ± 0.04 a	0.38 ± 0.04 a	0.38 ± 0.08 a
**20% WC**	0.49 ± 0.06 a	0.48 ± 0.05 a		
**10% WC**	0.44 ± 0.02 a	0.38 ± 0.04 b		
